# Prognostic impact of a senescence gene signature in multiple myeloma

**DOI:** 10.1007/s11357-025-01622-9

**Published:** 2025-03-25

**Authors:** Andrea Lehoczki, Otilia Menyhart, Hajnalka Andrikovics, Monika Fekete, Csaba Kiss, Gabor Mikala, Zoltan Ungvari, Balázs Győrffy

**Affiliations:** 1 Doctoral College, Health Sciences Division, Semmelweis University, Budapest, Hungary; 2 Department of Bioinformatics, Semmelweis University, 1094 Budapest, Hungary; 3 Cancer Biomarker Research Group, Institute of Molecular Life Sciences, Hungarian Research Network, Magyar Tudósok Körútja 2, 1117 Budapest, Hungary; 4 National Laboratory for Drug Research and Development, Magyar Tudósok Körútja. 2. 1117, Budapest, Hungary; 5 Healthy Aging Program, Institute of Preventive Medicine and Public Health, Semmelweis University, Budapest, Hungary; 6 Laboratory of Molecular Genetics, Central Hospital of Southern Pest, National Institute for Hematology and Infectious Diseases, Budapest, Hungary; 7 Department of Hematology and Stem Cell Transplantation, Central Hospital of Southern Pest, National Institute for Hematology and Infectious Diseases, Budapest, Hungary; 8 Vascular Cognitive Impairment, Neurodegeneration and Healthy Brain Aging Program, Department of Neurosurgery, University of Oklahoma Health Sciences Center, Oklahoma City, OK USA; 9 Stephenson Cancer Center, University of Oklahoma, Oklahoma City, OK USA; 10 Oklahoma Center for Geroscience and Healthy Brain Aging, University of Oklahoma Health Sciences Center, Oklahoma City, OK USA; 11 Department of Health Promotion Sciences, College of Public Health, University of Oklahoma Health Sciences Center, Oklahoma City, OK USA; 12 International Training Program in Geroscience, Doctoral College, Health Sciences Division/Institute of Preventive Medicine and Public Health, Semmelweis University, Budapest, Hungary; 13 Department of Biophysics, Medical School, University of Pecs, Pecs, 7624 Hungary

**Keywords:** Gene expression, Aging, Prognostic factors, Biomarkers

## Abstract

Multiple myeloma (MM), an incurable malignancy of plasma cells, is predominantly an age-related disease, with the majority of cases occurring in patients over the age of 60. Cellular senescence, a fundamental biological process underlying aging, has been increasingly recognized for its critical role in developing age-related malignancies. In this study, we aimed to investigate the prognostic significance of genes implicated in the molecular mechanisms of senescence within a large cohort of MM patients. Gene expression and clinical data from 1416 MM patients were obtained from four GEO datasets (GSE24080, GSE4204, GSE57317, and GSE9782) and integrated into a unified database. The raw data were processed using MAS5 normalization, scaling adjustments, and JetSet probe selection to ensure cross-platform comparability. A curated set of senescence-associated genes, the SenMayo gene signature, was employed for subsequent analyses. The final gene signature was computed as a weighted mean expression of 122 senescence-associated genes, with weights derived from univariate hazard ratios. Prognostic significance was evaluated using Cox regression, Kaplan–Meier survival analysis, and multivariate models incorporating clinical parameters such as gender, isotype, and molecular subtypes. False discovery rate (FDR) correction was applied to ensure the statistical robustness of findings. The weighted SenMayo gene signature strongly correlated with overall survival in MM patients (HR = 0.6, 95% CI = 0.47–0.76, *p* = 1.7e-05). The 75th percent probability of survival was reached at 36.1 months in the low-expression patient group, compared to 57 months in the high-expression group. Independent validation in datasets with sufficient patient numbers confirmed the prognostic value of the SenMayo signature (GSE4204: HR = 0.58, 95% CI = 0.39–0.88, *p* = 0.0089; GSE24080: HR = 0.61, 95% CI = 0.45–0.83, *p* = 0.0012; GSE57317: HR = 0.25, 95% CI = 0.08–0.77, *p* = 0.0095). Multivariate analyses further established the SenMayo signature as an independent prognostic factor, even when accounting for established clinical parameters such as sex and isotype. These findings underscore the robustness and independence of the SenMayo gene signature as a predictor of overall survival in multiple myeloma. This signature provides clinically valuable insights into the role of cellular senescence in disease progression.

**Supplementary Information:**

The online version contains supplementary material available at 10.1007/s11357-025-01622-9.

## Introduction

Multiple myeloma (MM) is an age-related hematologic malignancy characterized by the uncontrolled proliferation of plasma cells in the bone marrow, leading to immunodeficiency, bone destruction, and renal dysfunction [1]. MM represents a significant public health burden, accounting for approximately 1% of all cancers and 10% of hematologic malignancies worldwide [2]^.^ In 2022, an estimated 188,000 new multiple myeloma cases and 121,000 deaths occurred globally [3]. Incidence rates were among the highest in Northern America, exceeding 4 per 100,000 for both sexes combined, while mortality rates peaked at 1.8 per 100,000 in regions such as Northern Europe [3]. Its incidence rises sharply with age, with the majority of diagnoses occurring in individuals over 60 years old [1]. The expanding elderly population has contributed to an increasing prevalence of MM, underscoring the need for improved prognostic biomarkers and targeted therapeutic strategies [1].

The field of geroscience, which investigates the molecular mechanisms of aging as fundamental drivers of age-related diseases, offers a valuable framework for understanding MM pathogenesis [4]. Advances in geroscience have shed light on the critical role of biological aging mechanisms in the development of age-related diseases, including malignancies [1, 4]. Among these mechanisms, cellular senescence has emerged as a pivotal process linking aging with tumorigenesis [5, 6]. Cellular senescence is a complex biological phenomenon that induces stable cell cycle arrest, accompanied by profound alterations in gene expression, secretion of pro-inflammatory factors, and extracellular matrix-degrading enzymes known as the senescence-associated secretory phenotype (SASP) [7–9], and metabolic reprogramming [10, 11]. Senescent cells accumulate in various tissues with age, contributing to chronic inflammation and a tumor-permissive microenvironment [11]. While cellular senescence initially functions as a tumor-suppressive mechanism by preventing the uncontrolled proliferation of damaged cells, its paradoxical role in fostering a pro-inflammatory milieu and reshaping the tissue microenvironment suggests that increased susceptibility to senescence may either suppress or facilitate tumor progression and metastasis [5, 12–17]. Recent studies have highlighted the role of senescence-associated genes in various malignancies, including solid tumors [18, 19]. These findings suggest that identifying senescence-related gene expression patterns may offer insights into MM biology and prognosis [20–23].

Given the growing interest in senolytic and senostatic drugs, elucidating the role of senescence in MM could pave the way for new therapeutic strategies to improve patient outcomes. In this study, we aimed to investigate the prognostic significance of a senescence gene signature in MM patients. We utilized large-scale transcriptomic datasets and applied bioinformatics approaches to assess the association between senescence-related gene expression and overall survival. By leveraging the SenMayo gene signature, a curated set of senescence-associated genes validated across multiple tissues and species [24], we evaluated its prognostic potential in a cohort of 1416 MM patients. Our analyses included univariate and multivariate survival assessments to determine whether this gene signature serves as an independent prognostic factor in MM. Our findings contribute to the expanding body of evidence supporting the role of cellular senescence in age-related malignancies and highlight the potential of senescence-related biomarkers in prognostic stratification and treatment planning for MM patients.

## Methods

### Database selection and cohort identification

Transcriptome-level gene expression datasets for multiple myeloma were searched in the Gene Expression Omnibus (GEO) repository (https://www.ncbi.nlm.nih.gov/geo/). Inclusion criteria required datasets to have at least 30 samples, include accompanying clinical data, and utilize the Gene Expression Omnibus platforms GPL96, GPL570, or GPL571. These platforms were specifically chosen due to their consistent measurement of 22,277 genes using identical probe sequences, ensuring uniform sensitivity, specificity, and dynamic range across all samples, aiming to minimize variability and maintain comparability across datasets.

### Data preprocessing and quality control

The raw gene expression data underwent multi-step preprocessing and quality control to ensure consistency and reliability. Each array was normalized using the MAS5 algorithm, which performs well compared to RT-PCR-validated expression values [25]. The method allows for independent normalization of individual samples, ensuring that the inclusion or exclusion of any sample does not affect the integrity of the remaining dataset.

Subsequently, a scaling normalization adjusted the mean expression of the 22,277 overlapping probes to a uniform value of 1000 across all arrays, effectively reducing batch effects and ensuring comparability across datasets. Only probes in the GPL96 platform were used to maintain consistency, avoiding platform-specific biases introduced by the additional probes in GPL570 arrays [26].

The most reliable probe set for each gene was identified using the JetSet algorithm to refine the dataset further. Redundant samples characterized by identical expression values were removed, with only the first occurrence retained. Comprehensive quality control checks assessed background signal intensity, noise levels, and the percentage of present calls to evaluate signal reliability. Experimental consistency was monitored by detecting bioBCD spikes, while the 3′/5′ ratios of housekeeping genes GAPDH and ACTB were examined to ensure RNA integrity and sample quality. Samples that met all quality control criteria or fell within the 95% confidence interval for continuous variables were retained. Outliers, defined as samples failing one or more parameters, were excluded from further analyses. This robust preprocessing framework ensured high-quality, unbiased data for downstream analyses [27].

### Senescence signature and gene selection

To investigate the role of cellular senescence in multiple myeloma, we utilized the SenMayo gene set, a curated collection of senescence-associated genes validated across diverse tissues and species. The signature, initially established by Saul et al., encompasses genes consistently enriched in senescent cells and represents key pathways involved in the senescence program, including cell cycle arrest, senescence-associated secretory phenotype (SASP), DNA damage response, and mitochondrial dysfunction [24]. Each gene in the SenMayo set was mapped to its most reliable probe on the GPL96 platform using the JetSet algorithm to ensure accurate and specific expression measurements. Of the 125 genes in the original SenMayo-gene list, 122 were identified among the array probes; hence, all subsequent analyses were conducted on the selected 122 genes (Supplemental Table 1). The senescence signature was calculated as the weighted average expression of all included genes, with weights derived from univariate hazard ratios (HRs). Genes with HR > 1 were assigned a negative weight, reflecting their association with increased risk, while genes with HR < 1 received positive weights, indicating a protective role. This weighted approach allowed for a precise evaluation of the prognostic impact of senescence-associated pathways.

**Table 1:** Demographic and clinical characteristics of multiple myeloma patients in the integrated database. All percentages were calculated as the portion of the total number of patients (*n* = 1416). Not all data were available for all patients. Molecular subtypes: CD1 — cases with IgH translocations activating *CCND1* or *CCND3*; CD2 — IgH translocations activating *CCND1* or *CCND3*, and the expression of the early B-cell markers *CD20* and *PAX5*; HY, hyperdiploid subgroup; LB, low bone disease subgroup; MF — increased expression of *c-MAF* or *MAFB*; MS — upregulation of *FGFR3* and/or MMSET; MY, myeloid-like subgroup; PR, proliferation subgroup [32]. Abbreviations: Isotypes: FLC, Free Light Chain; IGA, Immunoglobulin A; IGG, Immunoglobulin G; Light Chain, monoclonal light chains without accompanying heavy chains

**Feature**		***n* (%)**
Included datasets		4
Total number of patients		1416
Survival		
	Median follow up	30 months
	Number of patients with an event	433 (30.6%)
Age (years)		58.2 (± 9.8)
Sex		
	Male	496 (35%)
	Female	327 (23%)
	No data	593 (42%)
Race		
	White	726 (51.2%)
	African American	22 (1.6%)
	No data	668 (47.2%)
Isotype		
	FLC	84 (5.9%)
	IGA	187 (13.2%)
	IGG	463 (32.7%)
	Light Chain	51 (3.6%)
Molecular subtypes		
	CD1	33 (2.3%)
	CD2	73 (5.2%)
	HY	127 (9%)
	LB	64 (4.5%)
	MF	39 (2.8%)
	MS	70 (4.9%)
	MY	127 9%)
	PR	60 (4.2%)
Treatment type		
	Dexamethasone	76 (5.3%)
	Total Therapy 2 (TT2)	345 (24.4%)
	Total Therapy 3 (TT3)	214 (15.1%)
	PS341 (bortezomib)	188 (13.3%)
	Total Therapy 6 (TT6)	55 (3.9%)
	Previously untreated	538 (38%)

### Univariate survival analysis

Univariate Cox regression analysis was conducted to assess the prognostic significance of the SenMayo gene signature on overall survival (OS). The Kaplan–Meier plotter was utilized to analyze the association between gene expression and survival [28, 29]. The analysis encompassed the entire range of expression levels between the lower and upper quartiles to minimize potential bias from specific cutoff values. Then, false discovery rate (FDR) was controlled using the Benjamini–Hochberg method to account for multiple hypothesis testing [30, 31]. Kaplan–Meier survival plots were generated based on the identified optimal cutoffs, which visually represented the impact of gene expression on survival outcomes.

### Multivariate analysis

A multivariate Cox regression analysis was conducted to evaluate the combined effect of the senescence signature and key clinical parameters, including gender, isotypes, and molecular subgroups. Each analysis was performed as a paired model (e.g., the expression of the mean senescence signature and gender or the senescence signature and isotype in a single model) to reduce the influence of missing data within the clinical datasets.

## Results

### Database setup

The comprehensive, integrated myeloma database includes 1416 tumor samples with available overall survival and transcriptome-level gene expression data derived from four primary datasets: GSE24080 (*n* = 559), GSE4204 (*n* = 538), GSE57317 (*n* = 55), and GSE9782 (*n* = 264). These datasets utilized two microarray platforms: GPL570 (1152 patients) and GPL96 (264 patients). While overall survival data were available for all patients, not all clinical parameters were consistently recorded. At the time of the last follow-up, 983 patients were alive, with a median overall survival of 30 months (0.1–269 months). Relapse status was documented for 801 patients, with 44.6% experiencing a relapse.

Age data were available for 823 patients, with a mean age of 58.2 years (range, 24.8–86), similar between sexes (58.3 years in males and 57.9 years in females). Most patients were White, and 1.6% of patients were identified as African American. The integrated patient cohort was further stratified into four isotypes (Free Light Chain, Immunoglobulin A, Immunoglobulin G, and Light Chain) and eight molecular subgroups (CD1, CD2, HY, LB, MF, MS, MY, and PR) [32, 33]. Of the entire cohort, 38% of patients were previously untreated. Treatments for myeloma included dexamethasone, TT2, TT3, TT6, and bortezomib (PS341). The integrated myeloma dataset’s detailed demographic and clinical characteristics are presented in Table 1.

### Univariate survival analysis

**Fig. 1:**
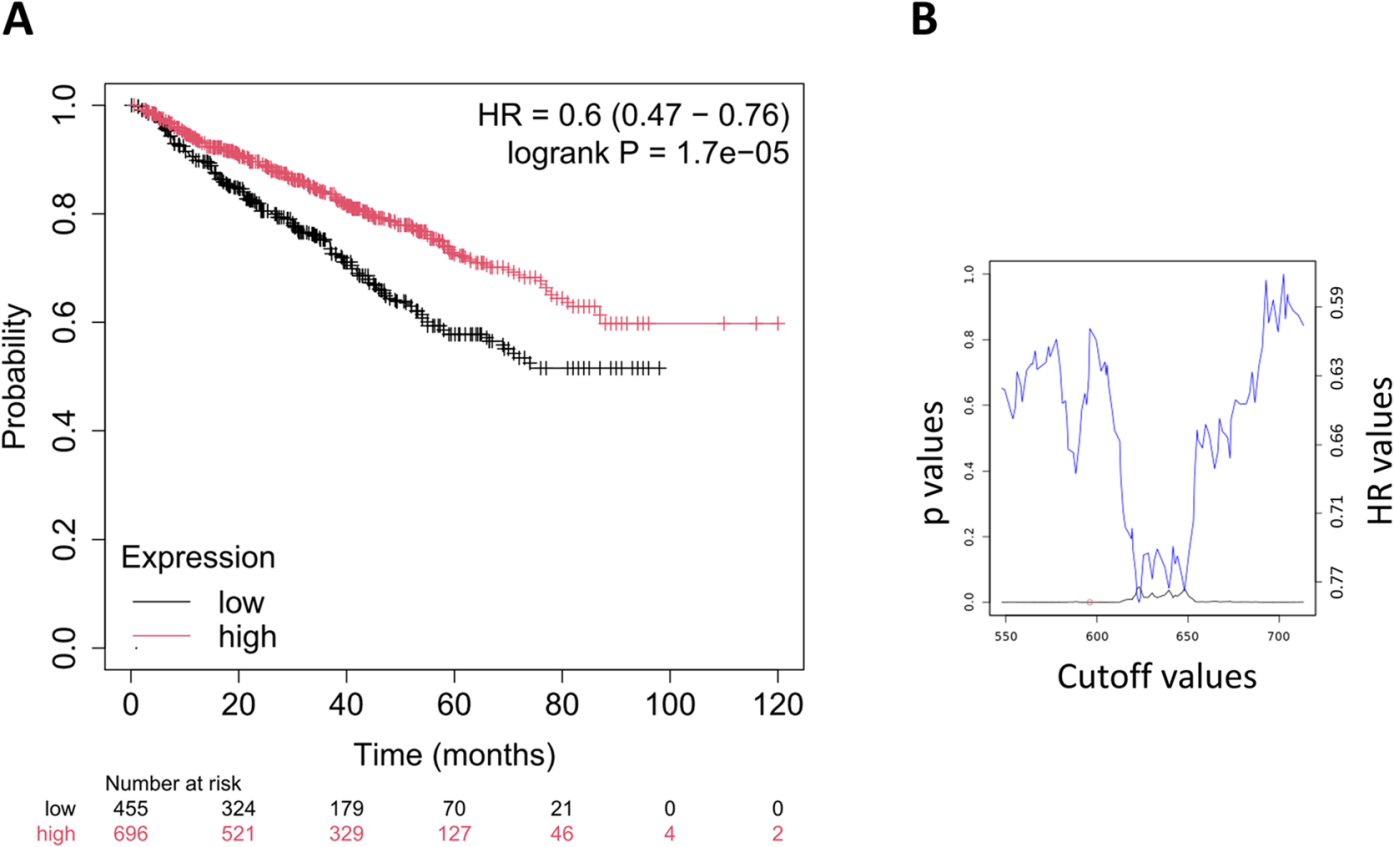
Prognostic significance of the SenMayo senescence gene signature in multiple myeloma. **A** Kaplan–Meier survival analysis demonstrates the association between the SenMayo gene signature and overall survival. Patients were stratified into low- and high-expression groups based on the weighted mean expression of 122 senescence-associated genes. **B** The robustness of the SenMayo signature was evaluated across various cutoff values. The optimal cutoff for survival stratification was identified at the point corresponding to the lowest *p*-value, as indicated by the red circle

We assessed the link between the weighted mean expression of the SenMayo gene signature, as described in the “Methods” section, and overall survival in multiple myeloma patients. The follow-up period for overall survival was censored at 120 months in all survival analyses. We identified a strong association with overall survival (HR = 0.6, 95% CI = 0.47–0.76, *p* = 1.7e-05; depicted in Fig. 1A), with a false discovery rate under 1%. The robustness of the SenMayo signature was evaluated across various cutoff values. The lowest *p*-value, highlighted with a red circle, was used to determine the optimal cutoff for gene expression stratification (Fig. 1B). In the low-expression patient group, the 75th percent probability of survival was 36.1 months. In contrast, it was 57 months in the high-expression group, demonstrating a significantly prolonged survival.

### Associations across independent datasets

**Fig. 2:**
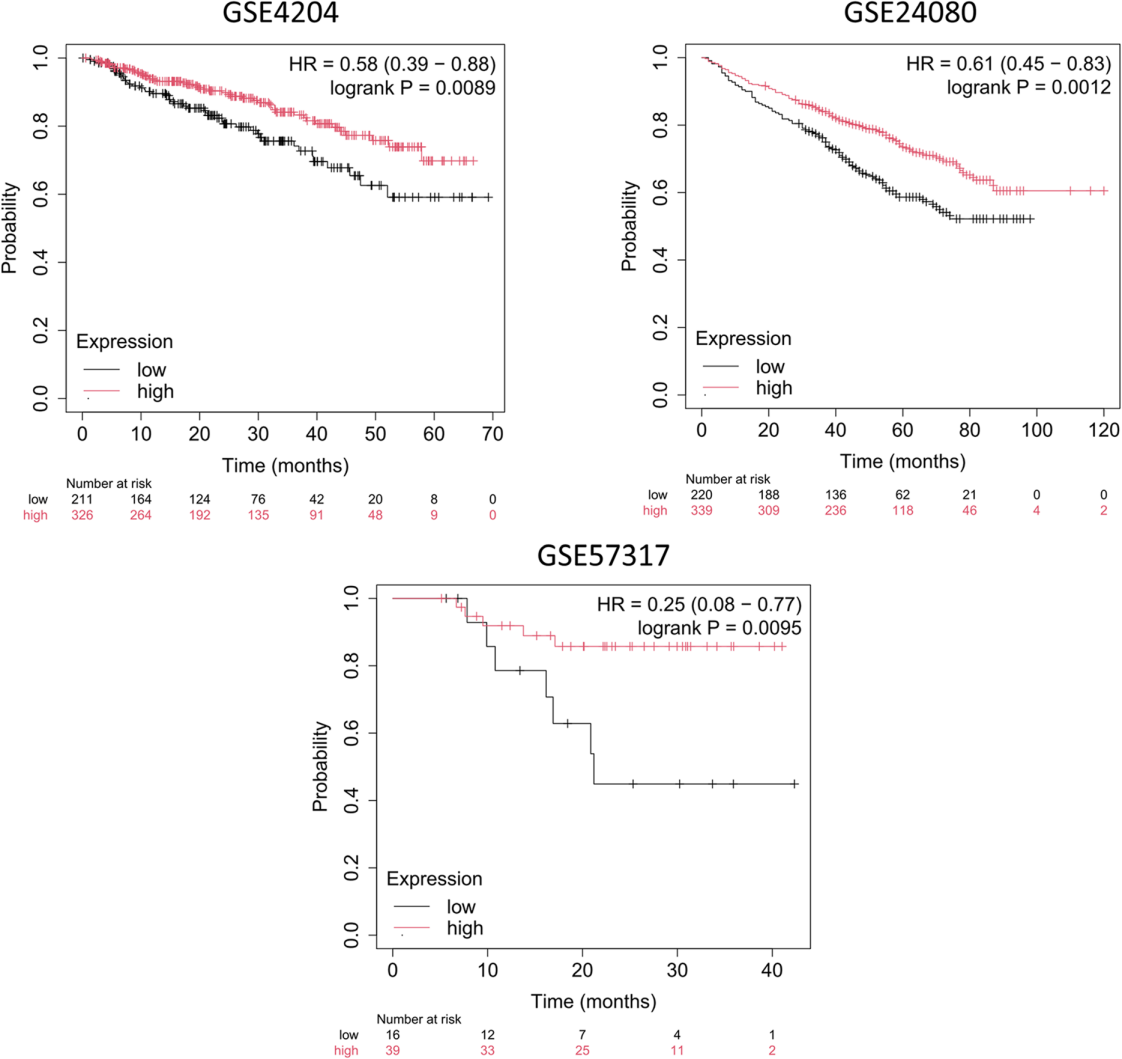
The association between overall survival and the senescence-derived gene signature remains significant when analyzing the prognostic power in three separate datasets. Note that because one dataset did not have sufficient cases for separate analysis, the total sample number does not equal the total sample number in Fig. 1*.* HR = hazard rate

To evaluate the overall prognostic value of the established senescence signature, we also conducted separate analyses in each dataset with sufficient patient numbers and available follow-up data. The weighted mean senescence signature demonstrated a significant association with survival in three independent datasets meeting our criteria (GSE4204, HR = 0.58, 95% CI = 0.39–0.88, *p* = 0.0089; GSE24080, HR = 0.61, 95% CI = 0.45–0.83, *p* = 0.0012; GSE57317, HR = 0.25, 95% CI = 0.08–0.77, *p* = 0.0095). In all three datasets, higher expression of the senescence gene signature was associated with worse overall survival outcomes. These findings indicate that the integrated signature exhibits a consistent and reproducible association with survival, regardless of the dataset used. Kaplan–Meier survival curves for each dataset are shown in Fig. 2.

### Multivariate survival analysis

To increase the sample size for multivariate analysis, we assessed the mean expression of the senescence-associated gene signature in combination with each available clinical parameter (sex, isotype, and molecular subtype) using separate models. The senescence signature demonstrated significant prognostic value when paired with isotype (*p* = 5.3e-13), which was also independently significant (*p* for isotype = 2.1e-04). While sex itself did not reach significance (*p* > 0.1), the senescence signature remained highly significant in this context (*p* = 1.8e-12). Of note, neither the molecular subtype nor the senescence signature achieves statistical significance when paired in a multivariate analysis. This outcome is likely attributable to the limited sample sizes within the molecular subgroups, which included only 6 to 27 non-censored patients with available survival data per subgroup. Such small cohorts are insufficient for a robust comparison between groups stratified by the senescence signature. Nevertheless, these findings suggest that the impact of the senescence signature may vary across molecular subtypes, warranting further investigation in a larger patient cohort. Overall, these findings indicate that the senescence signature retains its prognostic power when analyzed alongside specific clinical parameters, particularly isotype and sex.

## Discussion

Our study provides compelling evidence that the SenMayo senescence gene signature [24] is a significant prognostic factor in MM. The findings suggest that the senescence-associated gene expression profile is linked to disease progression and overall survival. By leveraging a large cohort of MM patients and employing robust bioinformatics analyses, we demonstrated that higher expression of senescence-associated genes correlates with improved survival outcomes. These results align with previous research underscoring the dual role of senescence in tumorigenesis, highlighting its tumor-suppressive and tumor-promoting effects depending on the cellular context [6, 11].

One of the key takeaways from our study is the complex interplay between senescence and MM progression. We assume that the higher mRNA expression of senescence-related genes reflects an increased presence of senescent cells in the MM samples. However, there is evidence that different cell types are affected by senescence in MM, further complicating the role of senescence in disease progression. While senescence limits the unchecked proliferation of malignant cells, SASP factors can contribute to a pro-inflammatory and immunosuppressive tumor microenvironment, potentially fostering disease progression [6, 11]. This dichotomy underscores the need for therapeutic strategies that selectively eliminate harmful senescent cells while preserving their tumor-suppressive properties. Senolytic and senostatic therapies have emerged as promising interventions, potentially mitigating the adverse effects of SASP while harnessing the protective aspects of senescence-induced growth arrest [34, 35]. Interestingly, while SenMayo expression was associated with differences in survival, it remains unclear whether this reflects variations in chemotherapy tolerance among these patients. Given that elderly MM patients often face treatment limitations due to frailty and comorbidities, it is possible that those with a high SenMayo signature possess better-preserved cellular resilience, enabling more effective chemotherapy administration. Future studies incorporating detailed treatment adherence and toxicity data will be essential to further investigate this possibility.

Beyond intrinsic senescence in MM cells, additional layers of complexity arise from therapy-induced senescence [36, 37], stromal senescence [23], and the senescence of other cell types within the tumor microenvironment [38]. Therapy-induced senescence, triggered by chemotherapeutic agents, can contribute to long-term treatment resistance by promoting the survival of senescent tumor cells that evade apoptosis. Similarly, senescence in the bone marrow stromal niche may play a critical role in shaping MM progression by modulating the supportive microenvironment that fosters plasma cell survival [22, 23]. Additionally, senescence of immune cells, including macrophages and T cells [39], could further impair anti-tumor immune responses, contributing to immune evasion and MM persistence.

MM is a highly heterogeneous disease that can be classified into distinct molecular subtypes based on genomic alterations, including chromosomal translocations, deletions, and copy number variations [40]. These alterations drive differential gene expression patterns, resulting in distinct biological pathways that may influence disease progression and treatment response. Molecular subtypes of MM, defined by cytogenetic abnormalities and transcriptomic profiles, include hyperdiploid MM, translocation-driven subtypes (such as t(4;14), t(11;14), and t(14;16)), and subgroups characterized by deletions (such as del(17p)). These molecular variations suggest that different MM subtypes may exhibit distinct pathways of tumorigenesis and differential interactions with senescence-associated processes. Given the substantial molecular heterogeneity of MM [41], it is likely that distinct molecular subtypes exhibit divergent senescence-associated pathways, potentially affecting their responses to therapy and disease progression. Despite this heterogeneity, our analysis did not reveal a statistically significant differential effect of SenMayo gene expression on survival across MM molecular subtypes. This may be due to the relatively small sample sizes within individual subtypes, limiting our ability to detect subtype-specific senescence-related survival differences. Future studies with larger cohorts and integrative multi-omics approaches could further elucidate how senescence-related pathways differentially influence MM progression in distinct molecular subtypes.

Our findings also have significant clinical implications. The ability to stratify MM patients based on senescence-associated gene expression could enable more precise prognostic assessments and inform personalized treatment strategies. Incorporating markers of senescence burden into existing prognostic models may improve risk stratification and guide therapeutic decision-making. Additionally, the identification of specific senescence-related pathways implicated in MM progression may open avenues for targeted interventions.

Senolytic treatments, which selectively target and eliminate senescent cells, have garnered significant interest in cancer therapy [42]. Notably, senescent cells exhibit heightened sensitivity to Bcl-2 inhibitors, a class of drugs that includes venetoclax [43–46]. Venetoclax has demonstrated remarkable efficacy in MM, particularly in patients harboring specific genetic subtypes such as the t(11;14) [47–49] and t(6;14) [50] translocations, which are associated with elevated Bcl-2 expression [51–53]. The landmark BELLINI study highlighted venetoclax’s ability to improve progression-free survival when used in combination with standard MM therapies [49]. However, its therapeutic benefit varies significantly across different molecular subtypes, underscoring the need to refine patient selection strategies to maximize efficacy. A comprehensive understanding of the interplay between senescence, Bcl-2 dependency, and molecular heterogeneity in MM is essential for optimizing senolytic therapies and tailoring effective treatment strategies. Given that distinct MM subtypes exhibit marked differences in sensitivity to Bcl-2 inhibitors, further investigations are needed to elucidate the mechanisms underlying this variability. Additionally, exploring the interplay between therapy-induced senescence and Bcl-2-targeted therapies may reveal novel strategies to enhance treatment efficacy. Future studies should also assess the potential of combining novel senolytic agents with existing MM treatment regimens to selectively eliminate senescent tumor cells while preserving immune function and minimizing adverse effects.

The SASP is a key mechanism through which senescent cells influence the tumor microenvironment [23, 38]. SASP factors include pro-inflammatory cytokines, chemokines, growth factors, and matrix-degrading enzymes, which can promote chronic inflammation, extracellular matrix remodeling, and immune suppression [54–57]. In MM, the bone marrow microenvironment plays a critical role in sustaining malignant plasma cells, and the presence of SASP-expressing senescent cells may further exacerbate disease progression by enhancing stromal support and suppressing anti-tumor immune responses [54–57]. While our study did not directly investigate SASP composition in MM, our findings underscore the importance of further research into the role of SASP-driven signaling pathways in MM pathophysiology. Targeting SASP components, in combination with senolytic therapies, may represent a novel strategy to disrupt the tumor-supportive microenvironment and improve patient outcomes.

These findings highlight several important directions for future research. A deeper understanding of the molecular mechanisms governing senescence in MM, particularly in the context of therapy-induced senescence and stromal senescence, will be essential for developing more effective therapeutic strategies. Investigating how senescence influences treatment response and resistance across MM subtypes may reveal novel therapeutic vulnerabilities. Moreover, exploring senescence-associated immune modulation could uncover new avenues for enhancing immune-based therapies in MM.

Despite the strengths of our study, several limitations warrant consideration. As our study is based on retrospective transcriptomic analyses, prospective validation in independent MM cohorts is necessary to confirm the robustness of our findings. Additionally, experimental studies evaluating the functional role of senescence-related genes in MM progression could provide mechanistic insights into their prognostic significance. Incorporating senescence biomarkers into clinical decision-making may improve patient stratification and refine therapeutic approaches in MM. Due to the lack of corresponding clinical data, we were unable to directly assess the correlation between SenMayo expression and patient age. However, prior studies have demonstrated that cellular senescence and the expression of senescence-associated genes generally increase with aging [58–60]. Future research with comprehensive age-related datasets would be valuable in further elucidating the relationship between age and the SenMayo signature in MM.

In conclusion, our study underscores the prognostic significance of the SenMayo senescence gene signature in MM and emphasizes the need for a nuanced approach to targeting senescence in MM therapy. While higher senescence-associated gene expression appears to confer a survival advantage, the broader implications of senescence in MM remain complex and multifaceted. Future investigations should focus on elucidating the differential roles of senescence across MM molecular subtypes, the impact of therapy-induced and stromal senescence, and the development of senescence-modulating therapeutic interventions to improve patient outcomes.

## Supplementary Information

Below is the link to the electronic supplementary material.

Supplementary file1 (XLSX 10 KB)
